# Sodium 4-phenylbutyrate ameliorates the effects of cataract-causing mutant gammaD-crystallin in cultured cells

**Published:** 2010-06-04

**Authors:** Bo Gong, Li-Yun Zhang, Dennis Shun-Chiu Lam, Chi-Pui Pang, Gary Hin-Fai Yam

**Affiliations:** Department of Ophthalmology and Visual Sciences, The Chinese University of Hong Kong, Hong Kong, China

## Abstract

**Purpose:**

gammaD-Crystallin (CRYGD) is a major structural lens crystallin and its mutations result in congenital cataract formation. In this study, we attempted to correct the altered protein features of G165fsX8 CRYGD protein with small chemical molecules.

**Methods:**

Recombinant FLAG-tagged mutants (R15C, R15S, P24T, R61C, and G165fsX8) of CRYGD were expressed in COS-7 cells and treated with small chemical molecules with reported protein chaperoning properties (sodium 4-phenylbutyrate [4-PBA], trimethylamine N-oxide [TMAO], and glycerol and DMSO [DMSO]). Protein solubility in 0.5% Triton X-100 and subcellular distribution was examined by western blotting and immunofluorescence, respectively. Apoptosis was assayed as the percentage of fragmented nuclei in transfected cells. Expression of heat-shock proteins (Hsp70 and Hsp90) was examined by reverse transcription-polymerase chain reaction analysis.

**Results:**

Unlike WT and most mutants (R15C, R15S, P24T, and R61C) of CRYGD, G165fsX8 CRYGD was significantly insoluble in 0.5% Triton X-100. This insolubility was alleviated by dose-dependent 4-PBA treatment. The treatment relieved the mislocalization of G165fsX8 CRYGD from the nuclear envelope. Also, 4-PBA treatment reduced cell apoptosis and caused an upregulation of Hsp70.

**Conclusions:**

4-PBA treatment reduced the defective phenotype of mutant G165fsX8 CRYGD and rescued the affected cells from apoptosis. This could be a potential treatment for lens structural protein and prevent lens opacity in cataract formation.

## Introduction

Protein aggregation and mistrafficking characterize many human disorders [1,2]. Protein folding to a native and functionally active state is often assisted by the concentrated milieu of cellular environment and folding machinery, including enzymes, molecular chaperones, pH regulators, ions and transporters as well as input of metabolic energy [3,4]. Conversely, they may misfold or unfold under stress conditions, including aging, pH/ion or temperature fluctuation, and genetic mutation. Protein quality control is a housekeeping machinery to correct aberrant proteins for proper folding or remove them through endoplasmic reticulum-associated degradation (ERAD) [5-8]. When protein folding stress overwhelms the protective action of quality control, aggregation could occur [9-11], which is recognized as a major cause of different pathological diseases. This not only confers loss-of-function, but also gain-of-function proteotoxicity or cytotoxicity. Mistrafficking of proteins could also trigger organelle instability or even dysfunction.

Recently, we identified G165fsX8 γD-crystallin (CRYGD) as a cause of congenital nuclear cataract in a Chinese family [12]. CRYGD is a structural protein essential for lens transparency. It exists as a highly symmetric monomer with four Greek key motifs organized into two highly homologous β-sheets connected by a six-residue linker. Premature truncated G165fsX8 mutation removed the last β-strand of the 4th Greek key motif and deleted Val170, a crucial interdomain residue for the intrinsic stability of native CRYGD. Hence, the mutant protein was prone to precipitate and loss of solubility. With the transfection in COS-7 cells, G165fsX8 CRYGD was misolocalized to the nuclear envelope, suggesting an impairment to the nuclear transfiguration in lens fiber cell differentiation, leading to opacity development [12]. Moreover, the transfected cells underwent apoptosis, which could also associate with lens cell defects in cataract formation. In this study, we tested if the mutant features and cellular defects could be amended by a small molecule chemical with chaperoning activity. Our result demonstrated a potential correction of mutated structural protein with a folding problem.

## Methods

### Expression constructs and mutagenesis

pFLAG/myc-CRYGD^WT^ and pFLAG-CRYGD^G165fs^ was prepared and sequences were confirmed previously [12]. Other CRYGD variant expression constructs were prepared and sequences were confirmed [13].

### Cell culture and transfection

COS-7 cells (American Tissue Culture Collection, Manassas, VA) were maintained in Eagle’s Minimal Essential medium (Invitrogen, Carlsbad, CA) with 10% fetal bovine serum (FBS; Invitrogen) and antibiotics. Prior to transfection, negligible endogenous *CRYGD* mRNA and protein of COS-7 cells was verified by reverse transcription-polymerase chain reaction (RT–PCR) and western blotting, respectively [12]. Cells (10^5^ cells/cm^2^) were transfected with *CRYGD* constructs by using FuGene HD reagent (Roche, Basel, Switzerland) at a ratio of 3 μl FuGene per μg DNA in Opti-MEM^®^I (Invitrogen). Chemical chaperone treatments were started 24 h after transfection.

### Treatment by chemical chaperones

Sodium 4-phenylbutyrate (4-PBA, 0.25 to 3 mM, triButyrate; Triple Crown America Inc., Perkasie, PA), trimethylamine N-oxide (TMAO, 25 to 300 mM; Sigma,  St. Louis, MO), glycerol (1 to 5%; Sigma) or DMSO (DMSO, 0.5 to 1%; Sigma) was added to the transfected cell culture. Fresh medium containing drugs was replenished every two days.

### Triton X-100 (Tx) solubility analysis

Cells were washed with ice-cold PBS and added with lysis buffer with 100 mM Tris-HCl (pH 7.4), 3 mM EGTA (Sigma), 5 mM MgCl_2_, 0.5% Tx (Sigma), protease inhibitor cocktail (Roche) and phenylmethylsulfonylfluoride 1 mM (PMSF; Sigma) for 2 min on ice. After centrifugation, clear supernatant containing Tx-soluble protein was denatured in buffer with 2% Na dodecylsulfate (SDS) and 50 mM _DL_-dithiothreitol (DTT; Sigma). The pellet containing Tx-insoluble protein was washed twice with ice-cold PBS, sonicated, and denatured in 9 M urea-SDS buffer. The samples were analyzed with SDS–PAGE and western blotting using horseradish peroxidase (HRP)-conjugated antibodies against FLAG (recognizing CRYGD), glyceraldehyde 3-phosphate dehydrogenase (GAPDH), or β-actin (Sigma), followed by enhanced chemiluminescence (ECL; GE Healthcare, Pittsburgh, PA). Band intensity was analyzed by Quantity One Image Analysis 4.6.2 (BioRad, Hercules, CA). FLAG expression was normalized with GAPDH for Tx-soluble protein and β-actin for Tx-insoluble protein. Statistical significance was determined by independent Student’s *t* test.

### Immunofluorescence

Cells were fixed with 2% neutral buffered paraformaldehyde in PBS, permeabilized and detected with mouse monoclonal anti-FLAG (recognizing CRYGD; Sigma) followed by appropriate fluorescence conjugated IgG secondary antibody (Jackson ImmunoRes Lab, West Gloves, PA) and DAPI (Sigma) staining.

### Transcription analysis

Total RNA was purified using a RNeasy kit (Qiagen, Valencia, CA) and an on-column RNase-free DNase kit (Qiagen). cDNA from 1 μg RNA, 10 ng/ml random hexanucleotide primer (Invitrogen) and reverse transcriptase (SuperScript III; Invitrogen) was amplified for heat-shock proteins, *Hsp70* (forward: 5'-AAG TAC AAA GCG GAG GAC G-3', reverse: 5'-GAT GGG GTT ACA CAC CTG C-3'), *Hsp90* (forward: 5'-ACC CAG ACC CAA GAC CAA CCG-3', reverse: 5'-ATT TGA AAT GAG CTC TCT CAG-3') and housekeeping *GAPDH* (forward: 5'-GAA GGT GAA GGT CGG AGT-3', reverse: 5'-GAA GAT GGT GAT GGG ATT TC-3'). After agarose gel electrophoresis, the specific band intensity was analyzed and normalized with housekeeping *GAPDH* expression. Statistical significance was assayed using independent Student’s *t* test.

### Terminal apoptosis assay

Paraformaldehyde-fixed cells were stained for FLAG and red X-conjugated secondary antibody and nuclei counterstained with DAPI. Samples were examined by fluorescence microscopy (DMRB; Leica, Wetzlar, Germany) equipped with a color imaging system (Spot RT; Diagnostic Instruments, Sterling Heights, MI). Terminal apoptosis rate was represented as the percentage of cells with fragmented nuclei. For each experiment (n=3), 10 random images (40× objective) were analyzed.

## Results

### 4-PBA improved the solubility of G165fsX8 CRYGD

**Figure 1:**
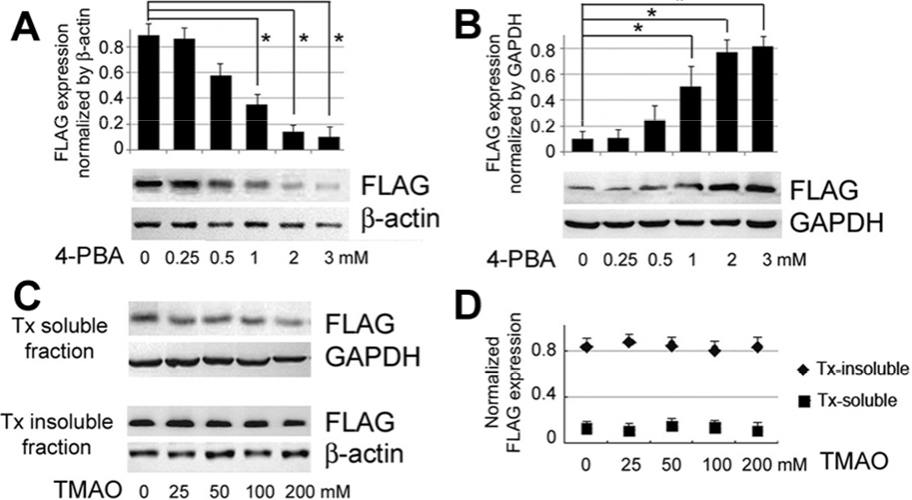
Chemical chaperone 4-PBA improved Tx solubility of G165fsX8 CRYGD expressing in COS-7 cells. Cells were treated with 4-PBA (0 to 3 mM) for two days followed by western blotting for FLAG-tagged CRYGD and band densitometry (represented by histogram). **A**: Tx insoluble fractions. **B**: Tx soluble fractions. β-Actin and GAPDH were the housekeeping proteins of Tx-insoluble and soluble fractions, respectively. Asterisks indicate a p<0.05 by independent Student’s *t*-test. **C**: Treatment of cells with TMAO (0 to 300 mM) for two days followed by Tx solubility test for FLAG-tagged CRYGD expression. **D**: Band densitometry analysis showed that TMAO did not affect the mutant protein solubility.

**Figure 2:**
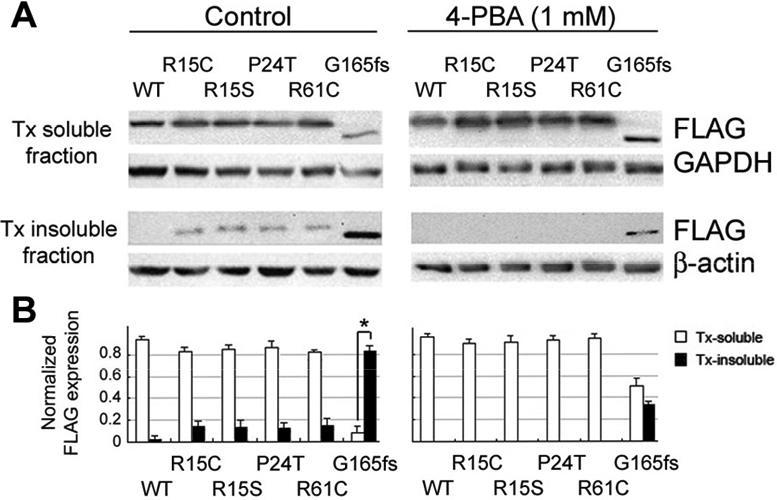
4-PBA improved the solubility of various cataract-causing CRYGD mutants expressing in COS-7 cells. **A**: western blotting of FLAG and housekeeping proteins in Tx soluble and insoluble fractions from cells expressing WT, R15C, R15S, P24T, R61C, or G165fsX8 CRYGD treated (right) or not (left) with 1 mM 4-PBA for two days. **B**: Band densitometry of FLAG-tagged CRYGD normalized with housekeeping proteins in cells from **A**. Asterisk indicated a p<0.05 by independent Student’s *t*-test.

The expected molecular size of FLAG/myc-tagged WT CRYGD was ~28 kDa and the size of truncated FLAG-tagged G165fs CRYGD (a shorten peptide and without myc) was ~24 kDa. We first confirmed our previous finding of the reduced Tx solubility of G165fsX8 CRYGD mutant in COS-7 cells (Figure 1). After western blotting of FLAG (representing CRYGD) followed by band densitometry analysis, we observed about 83% of truncated FLAG-tagged G165fsX8 CRYGD (~24 kDa) present in the Tx-insoluble fraction, whereas less than 5% FLAG/myc-tagged WT CRYGD (~28 kDa) was Tx-insoluble (Figure 2A) [12]. At one-day post-transfection, we started treating the cells with chemical chaperone 4-PBA (from 0.25 to 3 mM) for two days, followed by cell collection for Tx solubility assay. A dose-related change of Tx solubility of G165fsX8 mutant was observed (Figure 1A,B). Experiments were done in triplicate and treatment with 4-PBA from 1 to 3 mM caused a significant reduction of mean Tx insolubility to 37% (for 1 mM 4-PBA), 15% (2 mM 4-PBA) and 10% (3 mM 4-PBA), when compared to 83% of the untreated samples (p<0.05, independent Student’s *t*-test). Simultaneously, the amount of mutant protein in Tx soluble fractions increased proportionally (Figure 1B).

To validate this effect on other reported chemical chaperones, we treated the transfected cells in TMAO (from 25 to 200 mM) for 2 days but the results did not show any correction of Tx solubility (Figure 1C,D). Treatment with DMSO (0.5 to 1%) or glycerol (1 to 5%) was also ineffective in improving mutant solubility and cytotoxicity was observed (not shown). We confirmed that 4-PBA was more effective in lowering the amount of insoluble G165fsX8 GRYGD, to a level similar to that of WT protein.

### 4-PBA improved the solubility of different CRYGD mutants

To test if 4-PBA also improved the solubility of other reported cataract-causing CRYGD mutants (R15C, R15S, P24T and R61C), we created recombinant FLAG/myc-tagged CRYGD mutants by PCR-based site-directed mutagenesis and these constructs were confirmed by direct sequencing. These missense mutants with FLAG and myc tagging have an expected molecular size of ~28 kDa. An appreciable amount of mutant proteins were Tx-insoluble but the percentages were lower than that of G165fsX8 mutant (Tx insolubility of R15C: 17%, R15S: 15%, P24T: 14%, and R61C: 19%, compared to 83% for G165fsX8 and 5% for WT protein; Figure 2A). Following 4-PBA (1 mM) treatment for two days, the Tx insolubility of these missense mutants was reduced and most mutant protein became predominantly Tx-soluble (Figure 2). Only faint detection was noted in the Tx insoluble fractions. The reduction of G165fsX8 insolubility in 0.5% Tx was again noted after 4-PBA treatment.

### 4-PBA relocalized G165fs CRYGD from the nuclear envelope

**Figure 3:**
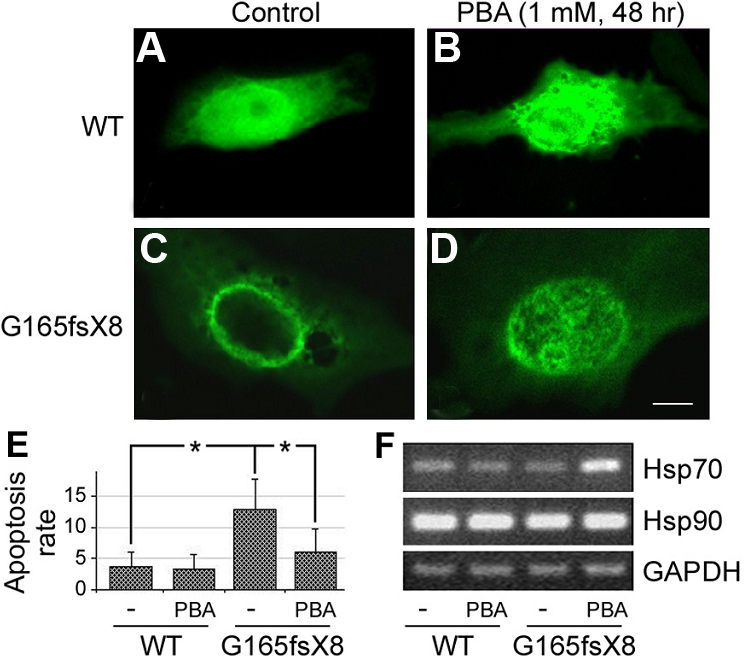
The corrective effect of 4-PBA on G165fsX8-expressing cells. **A**-**D**: 4-PBA corrected the mislocalization of G165fs CRYGD as shown by confocal immunofluorescence. **A**: Untreated WT cells and **B**: WT cells treated with 1 mM PBA for two days. **C**: Untreated G165fs CRYGD cells and **D**: G165fs CRYGD cells treated with 1 mM PBA for two days. **E**: 4-PBA reduced apoptosis of mutant cells with nuclear fragmentation. The asterisk indicates a p<0.05 by independent Student’s *t*-test. **F**: Semi-quantitative RT–PCR analysis showed specific upregulation of Hsp70 in G165fs CRYGD cells treated with 4-PBA (p<0.05, independent Student’s *t*-test). No significant change was found for Hsp90.

When expressed in COS-7 cells, G165fsX8 CRYGD was redistributed as a ring-shaped structure on the nuclear periphery (Figure 3C). Minimal staining in the cytoplasm and inner nucleus was observed. Our previous confocal microscopy study identified its colocalization with lamin A/C in the nuclear envelope [12]. In contrast, WT CRYGD was located in both nuclear and cytoplasmic regions (Figure 3A). This was different from the reported cytoplasmic staining of normal CRYGD and this could be due to the protein overexpression by cytomegalovirus (CMV) promoter. We focused our observation on the defective nuclear envelope staining of mutant CRYGD. Treatment with 4-PBA (1 mM) for five days successfully reduced the mutant cells showing CRYGD on the nuclear envelope. Instead, most treated cells exhibited nuclear and faint cytoplasmic CRYGD staining (Figure 3D). A quantitative analysis by counting a minimum of 200 trasnsfected cells in triplicated experiments showed that 20% of mutant cells were devoid of nuclear envelope staining of CRYGD after 0.5 mM 4-PBA treatment and 60% after 1 mM 4-PBA treatment. No change was found for WT CRYGD-expressing cells treated or not with 4-PBA (Figure 1A,B).

### 4-PBA rescued G165fsX8 mutant cells from apoptosis

In the same cell preparation and treatment, we quantified the apoptosis rate by the percentage of G165fsX8 CRYGD-expressing cells (FLAG-positive) showing fragmented nuclei upon DAPI staining. In untreated cells, the apoptosis rate was ~13%, which was about threefold higher than that of WT cells (4%; p<0.05, independent Student’s *t*-test; Figure 3E). Treatment with 1 mM PBA for five days significantly reduced the apoptosis rate to ~6% (p<0.05, independent Student’s *t*-test). There was no clear difference in the apoptosis rate of WT cells with or without 4-PBA treatment (Figure 3E).

### 4-PBA upregulated Hsp70 expression

We hypothesized that the correction of G165fsX8 CRYGD for greater solubility could be mediated through heat-shock responses initiated by 4-PBA. By RT–PCR analysis, the steady-state *Hsp70* and *Hsp90* expressions were investigated. Each experiment was done in triplicate and *GAPDH* amplification was the housekeeping control and normalization. We detected Hsp70 upregulation in mutant cells after 1 mM 4-PBA treatment (p<0.05, independent Student’s *t*-test; Figure 3F), but no change of *Hsp90* expression was observed.

## Discussion

γD-Crystallin (CRYGD) is a lens structural protein and its mutations are known to cause different types of congenital cataracts [12-16]. In this study, we studied different cataract-causing CRYGD mutants in vitro. While WT and most CRYGD mutants were mildly insoluble upon Tx extraction, G165fsX8 mutant demonstrated significant Tx insolubility. Unlike WT CRYGD, G165fsX8 was mislocalized to the nuclear envelope which was consistent with our previous report on its colocalization with lamin A/C [12]. Our present work attempted to rescue the disrupted features by treatment with small molecule chemicals with reported protein chaperoning activity. While TMAO, DMSO, and glycerol were not effective, 4-PBA was shown to reduce the Tx insolubility of G165fsX8 mutant dose-dependently and removed its localization from the nuclear envelope. This reduced the number of cells undergoing apoptosis. We also demonstrated upregulation of *Hsp70*, which could be involved in cell rescue.

Our study stated a proof-of-principle correction of lens *CRYGD* by 4-PBA to regain proper cellular features. This can be of therapeutic potential to alleviate, in our case congenital cataracts or, aggregopathies due to defective structural proteins. Chemical chaperoning has been shown experimentally to correct protein’s non-native conformation, mistrafficking and the associated cellular defects, resulting in cell survival [17-20]. Though the exact mechanisms are not well defined, chemical chaperones likely shift the folding equilibrium toward more native states, reduce non-productive aggregation or enhance the resident chaperoning environment. This improves protein folding and facilitates the transport of proteins across intracellular compartments. Increasing evidence has rendered the chaperone-assisted protein rescue an appealing strategy for protein folding disease management [21-34].

4-PBA is known as a peroxisome proliferator and histone deacetylase inhibitor to affect transcription [35-37]. It is approved for clinical use to restore chloride conductance in cystic fibrosis patients and treat urea cycle disorders [38-40]. No drug-induced toxicity was encountered by patients. Functioning as a chemical chaperone, it significantly improves the folding of different proteins, like α1-antitrypsin, nephrin, myocilin, Pael receptor and restores protein localization and cell viability [22,27,41,42]. The molecular mechanism of how 4-PBA recovers protein appears inconclusive. Though it has been reported as a transcriptional regulator, no significant gene changes were observed for the ER unfolded protein response [43]. In contrast, down-regulation of constitutive Hsc70 protein caused by the decreased stability of mRNA was observed, indicating a mode of action different from that of inhibiting deacetylase activity [44]. In our assay, we found an inducible Hsp70 expression after 4-PBA treatment. This could be beneficial for protein disaggregation, protein folding and complex remodeling, trafficking and regulation of heat-shock responses [45-49]. However, the role of Hsp70 in the folding of structural proteins is unknown, except the co-staining of Hsp70 with misfolded keratin in Mallory bodies [50]. Interaction of small Hsp47 with type I collagen was important for the Golgi transport [51,52]. Moreover, Hsp70 is anti-apoptotic by directly associating with Apaf-1, by antagonism of apoptosis-inducing factor, or through direct suppression of downstream caspases [53,54]. Inhibition of its function is sufficient to induce cell death in some tumors [55]. In zebrafish lens development, inducible Hsp70 is a vital regulator for lens fiber cell differentiation [56,57].

The result of 4-PBA correction of cells with G165fsX8 CRYGD was similar to that previously described for TMAO on G98R CRYAA [31]. However, TMAO was not effective for G165fsX8 CRYGD-expressing cells, the same as for 4-PBA on cells with G98R CRYAA. This indicates that mutations of different crystallin molecules may have specific chaperone treatments with unknown mechanisms. Though Hsp70 was upregulated after both treatments, TMAO has an additional role on the hydration energy in the protein folding process [58]. The presence of exposed hydrophobic patches in G98R CRYAA could thus be modified or removed by TMAO, resulting in a more stabilized conformation [31].

In conclusion, 4-PBA reversed the defective cellular features caused by G165fsX8 CRYGD and reduced apoptosis possibly through initiation of heat shock response. Hsp70 upregulation could be caused by 4-PBA directly or a change of chaperoning capacity in cells. It could be a consequence of mutant protein stabilization. Further work warrants a better characterization of Hsp70 regulation in cataractogenesis and for potential therapeutic strategy.
